# Possible prognostic impact of PKCι genetic variants in prostate cancer

**DOI:** 10.1186/s12935-023-03182-4

**Published:** 2024-01-10

**Authors:** Amna Hafeez, Maria Shabbir, Khushbukhat Khan, Janeen H. Trembley, Yasmin Badshah, Sameen Zafar, Kanza Shahid, Hania Shah, Naeem Mahmood Ashraf, Arslan Hamid, Tayyaba Afsar, Ali Almajwal, Afifa Marium, Suhail Razak

**Affiliations:** 1 Department of Healthcare Biotechnology, Rahman School of Applied Biosciences, National University of Sciences and Technology, Atta-Ur, Islamabad, Pakistan; 2 Department of Laboratory Medicine and Pathology, University of Minnesota, Minneapolis, MN USA; 3 Masonic Cancer Center, University of Minnesota, Minneapolis, MN USA; 4 Minneapolis VA Health Care System Research Service, Minneapolis, MN USA; 5 Department of Biochemistry and Biotechnology, University of Gujrat, Hafiz Hayat Campus, Gujrat, 50700 Punjab Pakistan; 6 University of Bonn, LIMES Institute (AG-Netea), Carl-Troll-Str. 31, 53115 Bonn, Germany; 7 Department of Community Health Sciences, College of Applied Medical Sciences, King Saud University, Riyadh, Saudi Arabia

**Keywords:** PKCι, Prostate cancer, Pathways, Molecular dynamic simulations

## Abstract

**Background:**

Single nucleotide polymorphisms (SNPs) have been linked with prostate cancer (PCa) and have shown potential as prognostic markers for advanced stages. Loss of function mutations in PKCι have been linked with increased risk of malignancy by enhancing tumor cell motility and invasion. We have evaluated the impact of two coding region SNPs on the PKCι gene (*PRKCI*) and their prognostic potential.

**Methods:**

Genotypic association of non-synonymous PKCι SNPs rs1197750201 and rs1199520604 with PCa was determined through tetra-ARMS PCR. PKCι was docked with interacting partner Par-6 to determine the effect of these variants on PKCι binding capabilities. Molecular dynamic simulations of PKCι docked with Par-6 were performed to determine variant effects on PKCι protein interactions. The possible impact of changes in PKCι protein interactions on epithelial cell polarity was hypothesized.

**Results:**

PKCι rs1199520604 mutant genotype TT showed association with PCa (p = 0.0055), while rs1197750201 mutant genotype AA also showed significant association with PCa (P = 0.0006). The binding interaction of PKCι with Par-6 was altered for both variants, with changes in Van der Waals energy and electrostatic energy of docked structures.

**Conclusion:**

Genotypic analysis of two non-synonymous PKCι variants in association with PCa prognosis was performed. Both variants in the PB1 domain showed potential as a prognostic marker for PCa. In silico analysis of the effect of the variants on PKCι protein interactions indicated they may be involved in PCa progression through aberration of epithelial cell polarity pathways.

**Supplementary Information:**

The online version contains supplementary material available at 10.1186/s12935-023-03182-4.

## Background

Prostate cancer (PCa) is the second most diagnosed cancer in men with 1.4 million new cases worldwide and the fifth leading cause of cancer related deaths with 375,000 deaths in 2020 alone. In Pakistan, 4550 new PCa cases and 2,188 deaths were registered in 2020 according to WHO [1]. The non-modifiable risk factors of prostate cancer, including familial history, gender, age, ethnicity and genetic factors, makes mitigating the incidence rates a difficult task [2]. Therefore, identifying better diagnostic, prognostic, and therapeutic avenues for PCa is the need of the hour. Genome wide association studies have identified greater than 100 SNPs in the human genome that can be associated with prostate cancer [3]. One of the genes that plays a role in PCa progression is protein kinase C iota (*PRKCI*) encoding PKCι ( alternate name KPCI), which is an atypical protein kinase belonging to the PKC family of kinases [4, 5]. Polymorphisms located in non-coding regions of *PRKCI* have been linked with PCa in different populations, however no SNP in the coding region has been reported in relation to PCa [6]. In this study, the non-synonymous variations rs1197750201 F66Y and rs1199520604 G34W, located in the PB1 domain of the PKCι regulatory region were selected for analysis. The aim was to investigate the association of rs1197750201 and rs1199520604 with PCa and to determine their potential as a prognostic marker. The effect of rs1197750201 F66Y and rs1199520604 G34W on protein–protein interaction of PKCι with Par-6, one of the main interacting partners of PKCι involved in establishment and maintenance of cellular polarity [7], was also investigated. Par-6 is involved in asymmetric cell division, cellular migration, and cell fate determination [8].

## Methods

### Study approval and sample collection:

Ethical approval for the study from institutional review board (IRB No. 10-2021-01/01) was obtained from parent department Atta-ur-Rahman School of Applied Biosciences (ASAB) of National University of Sciences and Technology. Blood samples from PCa patients (Combined Military Hospital (CMH), Rawalpindi, Pakistan) and healthy controls were collected for genotyping to determine the presence of the *PRKCI* variants. Written and oral informed consent were obtained from each participant before the collection of blood samples. PCa patients over the age of 50 years were included in this study. The samples were divided into two groups, i.e., tumors from stage 1–2 and 3–4. Patients with comorbidities were excluded. Patients having PCa as a secondary tumor were also excluded from the study. The clinical and pathological features of the patients are shown in (Table 1).

**Table 1:** Clinical and pathological features of PCa patients

Clinical-pathological features of patients	Number of patients (N) (%)
Age	
≤ 50	0
> 50	100
Stage	
I–II	51 (51)
III–IV	49 (49)
Metastasis	
Metastatic	55 (55)
Non- metastatic	45 (45)
Treatment	
Pre-treated	90 (90)
Treatment naïve	10 (10)

### PKCι variant selection

In a study conducted in 2022, different PKCι missense variants were evaluated for pathogenicity and potential for carcinogenesis [9]. Out of those variants, two were present in the PB1 domain were selected for this particular study because the function of the PB1 domain is essential for the interaction of PKCι with Par-6 during cellular polarity.

### Primer design

The primers for the Tetra ARMS PCR were designed using the tool Primer 1 [10] shown in Table 2. Two internal and two external primers were designed for the *PRKCI* genotyping and investigating the presence of selected missense polymorphisms in the control and patient data (Additional file 1: Table S1). The primers were validated using the UCSC in silico PCR [11]. Primer optimization was done to establish the conditions required for amplification of *PRKCI*.

**Table 2:** Genotype analysis of rs1199520604 PRKCI G34W and rs1197750201 PRKCI F66Y

Genotype	Patient (n = 100)	Control (n = 100)	Odds Ratio	95% CI Odds Ratio	Relative risk	95% CI Relative risk	P value
	n. (%)	n. (%)					
rs1199520604 PRKCI G34W							
GG	36 (36%)	56 (56%)	0.4222	0.2422 to 0.7467	0.6483	0.4771 to 0.8630	0.0031
TT	6 (6%)	20 (20%)	0.2609	0.1036 to 0.6537	0.4314	0.2039 to 0.8024	0.0055
GT	58 (58%)	24 (24%)	4.373	2.411 to 8.041	1.987	1.510 to 2.646	< 0.0001
G	65 (65%)	68 (68%)	0.8739	0.4834 to 1.568	0.9356	0.7093 to 1.264	0.7646
T	35 35%	32 (32%)	0.2534	0.1448 to 0.4642	0.5071	0.3709 to 0.6799	< 0.0001
rs1197750201 PRKCI F66Y							
TT	57 (57%)	24 (24%)	4.198	2.317 to 7.703	1.947	1.482 to 2.586	< 0.0001
AT	8 (8%)	16 (16%)	0.4565	0.1929 to 1.100	0.1264	0.3390 to 1.048	0.1264
AA	35 (35%)	60 (60%)	0.3590	0.2020 to 0.6431	0.5951	0.4352 to 0.7978	0.0006
A	39 (39%)	68 (68%)	0.3009	0.1658 to 0.5457	0.5557	0.4124 to 0.7377	< 0.0001
T	61 (61%)	32 (32%)	3.324		1.800	1.355 to 2.425	< 0.0001

### DNA Extraction and Genotyping

DNA extraction was performed through the phenol–chloroform method from the diseased and control samples [12]Tetra Amplification Refractory Mutation System Polymerase Chain Reaction (Tetra ARMS PCR) was performed for identification of the presence of SNP in the samples. Solis Biodyne FIREpol master mix was used for the PCR reaction in the Applied Biosystems™ Veriti™ 96-Well Thermal Cycler. Statistical analysis was performed using GraphPad Prism version 9.3.1 for Windows (GraphPad Software, San Diego, California USA, www.graphpad.com). Fisher’s exact test was applied to the data to evaluate the significance of association between the mutated *PRKCI* and prostate cancer. Genotype analysis with regards to metastasis and stage were also performed to get a comprehensive picture of the link between the chosen polymorphisms and PCa.

### Structure prediction and In situ mutagenesis

Due to the unavailability of PKCι tertiary structure in Protein Data Bank, protein structure prediction was performed through I-TASSER (Iterative Threading ASSEmbly Refinement) [13]. The model with the highest confidence score was selected for further analysis. The predicted structure was visualized using PyMOL molecular visualization system [14]. Previously determined structures of PKCι deposited in RCSB Protein Data Bank were searched for comparison with the predicted structure. The complete isolated structure of PKCι has not yet been deposited, however the structure of the kinase domain was determined (PDB Id:1ZRZ [15], PDB Id: 3A8X [16]) and the structure of the PB1 domain in complex with Par-6 alpha (PDB Id: 1WMH [17]) was also found. The structure predicted by I-TASSER was further validated through domain information generated by InterPro and cross-referenced through literature. In situ mutagenesis was performed using PyMol wizard tool to obtain PKCι-G34W and PKCι-F66Y variant structures for further analysis.

### Effect of polymorphisms on RNA stability

The effect of missense mutations G34W and F66Y on PKCι mRNA secondary structure stability were evaluated through the RNAFold software [18] based on thermodynamic parameters. The results were used to predict the effect of change in the mRNA secondary structure stability on PKCι protein structure.

### Effect of polymorphisms on protein–protein docking

In order to observe the effect of the chosen SNPs on protein interactions, docking of both PKCι wild-type and mutants was done with the interacting partner Par-6 through HADDOCK 2.4 [19]. The predicted docked structures with the lowest z-score obtained through HADDOCK 2.4 were chosen and the results were visualized through LigPlot + [20].

### Molecular dynamic simulation

The docked structure of Par-6 with PKCι wild-type, PKCιG34W variant and PKCι-F66Y variant were evaluated for their stability through molecular dynamic simulations in GROMACS 2018 [21] using OPLS-AA force field. A cubic box simulating a unit cell was solvated by adding water molecules SPC216 and Na + /Cl- ions were added to neutralize the net charge. Energy minimization of the system was performed utilizing 50,000 steps which was followed by system equilibration for NVT (Number of particles, Volume and Temperature) and NPT (Number of particles, Volume and Temperature). The same random seed was used for initiating the trajectories and the docked structures were simulated for 10 ns and the trajectory coordinates were saved every 10 ps. Analysis of the simulation was performed by using GROMACS built in program gmx_trjconv for building trajectories. GROMACS commands were used for calculating the root mean square deviations (RMSD), radius of gyration (Rg), solvent accessibility surface area (SASA) and the number of hydrogen bonds. The results of the simulations were visualized using the 3-dimensional structure Representation Sharing (3dRS) application [22].

### Pathway construction

Protein–protein interactions of PKCι were studied using databases including Reactome, Kyoto Encyclopedia of Genes and Genomes (KEGG), Nature Pathway Interaction Database (PID) and STRING and a pathway was constructed to illustrate the role of PKCι and Par6 in cell polarity.

## Results

### Association of PKCΙ variants rs1197750201 and rs1199520604 with PCa

For the PKCι variant rs1199520604 (G34W), statistical analysis (Table 2) revealed that the variant genotype GT had the most significant association with an increased risk of PCa (p = 0.0001), odds ratio = 4.3 (95% CI OR: 2.411 to 8.041), relative risk = 1.987 (95% CI RR: 1.510 to 2.646). The genotypes GG (wild) and TT (mutant) were also found to be significantly associated with PCa with p values 0.003 and 0.005 respectively. For PKCι variant rs1197750201 (F66Y), the wild-type genotype TT was most significantly associated with an increased risk of PCa (p ≤ =  < 0.0001, odds ratio = 4.198 (95% CI OR: 2.317 to 7.703), relative risk = 1.947 (95% CI RR: 1.482 to 2.586). The mutant genotype AA was associated with a decreased risk of PCa with a p = 0.0006, odds ratio = 0.359 (95% CI OR: 0.2020 to 0.6431), relative risk = 0.0.126 (95% CI RR: 0.4352 to 0.7978).

Genotype analysis in terms of metastatic and non-metastatic PCa with regards to rs1199520604 (G34W) showed no significant results, while the rs1197750201 (F66Y) wild-type genotype TT was significantly associated with increased risk of metastatic PCa (p = 0.01, odds ratio = 3.16 (95% CI OR: 1.364 to 7.847), relative risk = 2.011 (95% CI RR:1.173 to 3.641) while the mutant genotype AA showed a probable protective effect with the relative risk of 0.42 (95% CI RR: 0.1983 to 0.8233) and odds ratio of 0.26 (95% CI OR: 0.09588 to 0.7488) (p = 0.011). Analysis of genotype with regards to cancer stage for the rs1199520604 (G34W) and rs1197750201 (F66Y) did not yield significant results (Table 3).

**Table 3:** PRKCI genotype analysis according to metastasis and PCa stage

Metastatic analysis									Analysis according to cancer stage						
Genotype		Metastatic (n = 44)	Non-Metastatic (n = 56)	Odds Ratio	95% CI Odds Ratio	Relative Risk	95% CI Relative risk	P value	Stage 1–2 (n = 39)	Stage 3–4 (n = 61)	Odds Ratio	95% CI Odds Ratio	Relative Risk	95% CI Relative risk	P value
		n. (%)	n.(%)						n. (%)	n.(%)					
GG	PRKCI rs1199520604 G34W	10 (22.73%)	28 (50%)	0.2941	0.1186 to 0.6815	0.4799	0.2630 to 0.8185	0.0069	14 (35.90%)	21 (34.42%)	1.067	0.4482 to 2.415	1.040	0.6125 to 1.693	> 0.9999
GT		30(68.18%)	24 (42.86%)	2.578	1.117 to 5.905	1.714	1.078 to 2.828	0.0271	22 (56.41%)	34 (55.74%)	1.028	0.4724 to 2.252	1.017	0.6254 to 1.682	> 0.9999
TT		4 (9.09%)	4 (7.14%)	1.300	0.3595 to 4.677	1.150	0.4823 to 1.973	0.7281	3 (7.69%)	6 (9.84%)	0.7639	0.1998 to 2.859	0.8426	0.2972 to 1.757	> 0.9999
G		25 (52.17%)	40 (55.56%)	0.8727	0.4247 to 1.797	0.9205	0.5878 to 1.450	0.8499	25 (64.10%)	38 (62.30%)	1.081	0.4855 to 2.547	1.049	0.6414 to 1.785	> 0.9999
T		19 (47.83)	16 (44.44%)	1.900	0.8517 to 4.391	1.411	0.9019 to 2.158	0.1443	14 (35.90%)	23 (37.70%)	0.9252	0.3926 to 2.060	0.9535	0.5603 to 1.559	> 0.9999
TT	PRKCI rs1197750201 (F66Y)	34 (77.27%)	29 (51.79%)	3.166	1.364 to 7.847	1.997	1.173 to 3.641	0.0121	18 (46.15%)	25 (40.98%)	1.234	0.5674 to 2.671	1.136	0.6917 to 1.843	0.6807
AT		4 (9.09%)	6 (10.71%)	0.8333	0.2520 to 3.011	0.9000	0.3691 to 1.663	> 0.9999	2 (5.13%)	8 (13.12%)	0.3581	0.07379 to 1.639	0.4865	0.1354 to 1.304	0.3080
AA		6 (13.64%)	21 (37.50%)	0.2632	0.09588 to 0.7488	0.4269	0.1983 to 0.8233	0.0118	19 (48.72%)	28 (45.90%)	1.120	0.5216 to 2.401	1.071	0.6546 to 1.745	0.8388
A		36 (81.82%)	24 (42.86%)	0.2250	0.07353 to 0.8497	3.000	1.646 to 5.875	< 0.0001	19 (48.72%)	32 (52.46%)	0.8609	0.4003 to 1.877	0.9127	0.5582 to 1.489	0.8378
T		8 (18.18%)	32 (57.14%)	0.06622 to 0.4137	0.1180 to 0.7432	0.3333	0.1702 to 0.6075	0.00100	20 (51.28%)	29 (47.54%)	1.162	0.5328 to 2.498	1.096	0.6714 to 1.792	0.8378

### Effect of rs1197750201 and rs1199520604 polymorphisms on mRNA secondary structure

**Fig. 1:**
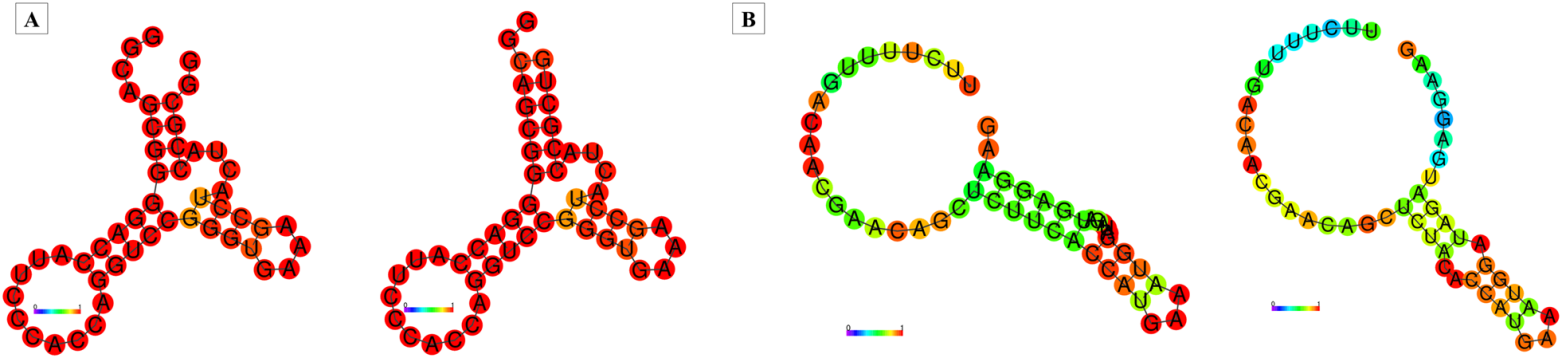
Effect of PKCι variants of RNA secondary structure. The lighter to darker shades of nucleotide represent base pair probabilities from 0 to 1. **A** Comparison of MFE structure drawing encoding base-pair probabilities between wild type and rs1199520604. **B** Comparison of MFE structure drawing encoding base-pair probabilities between wild type and rs1197750201

The comparison of the mRNA secondary structure produced by a stretch of 50 nucleotides including the position for the SNP rs1199520604 showed that the wild-type RNA had minimum free energy (MFE) of -16.80 kcal/mol while the mutant had -19.80 kcal/mol, meaning it was more stable due to lowered MFE. A similar comparison for rs1197750201 yielded -6.20 kcal/mol for the wild type allele while a minimum free energy of -3.70 kcal/mol for the mutant, meaning the variant structure was less stable in comparison to wild-type Fig. 1.

### Effect of PKCι variants on protein–protein interaction

**Fig. 2:**
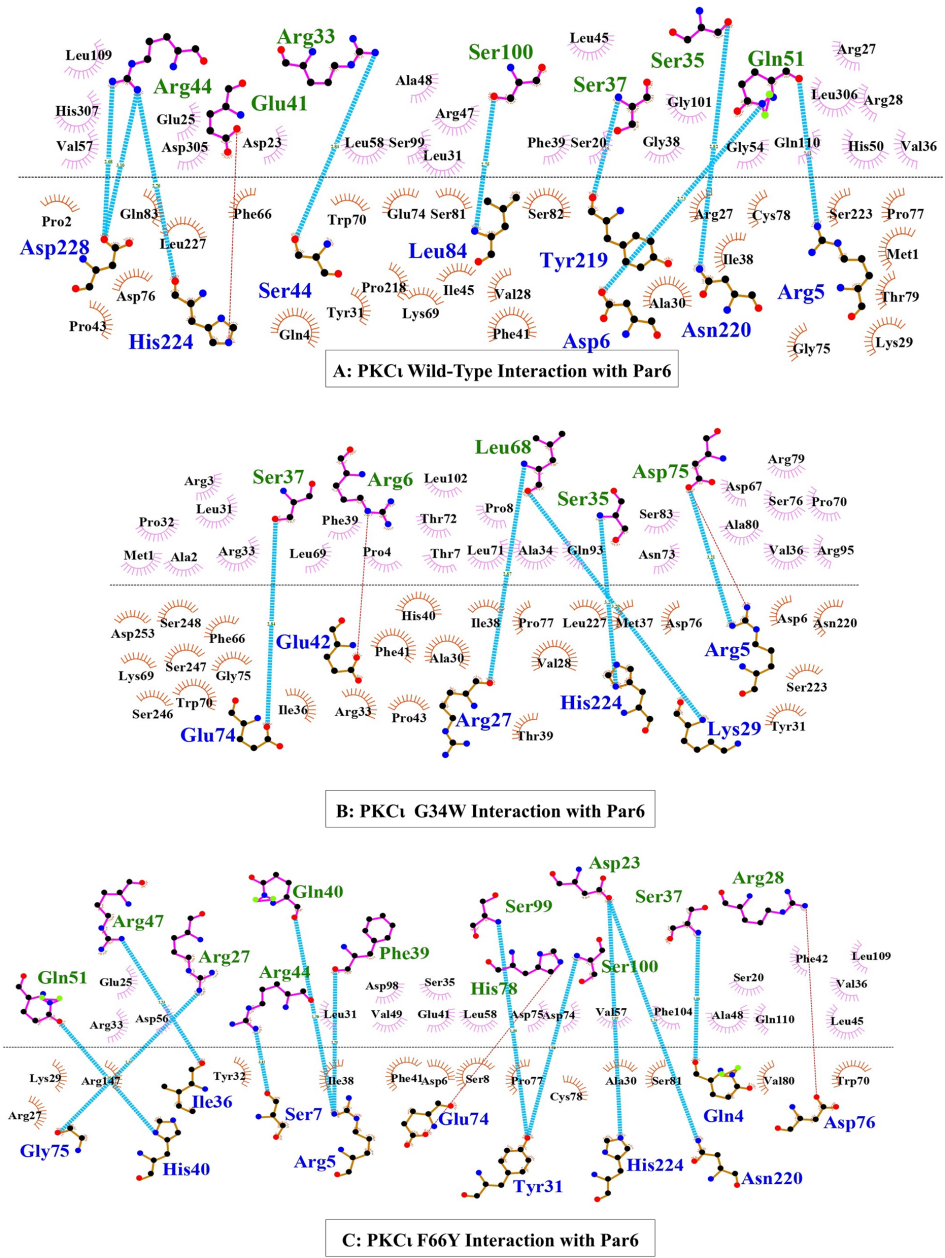
Comparison of PKCι wild-type, PKCι-G34W and PKCι-F66Y docked with Par-6. The amino acids below interface belong to PKCι and the amino acid above belong to Par-6. The curved lash structures represent hydrophobic interactions between the amino acids of the two docked proteins. The blue lines represent hydrogen bonds while the red lines represent salt bridges

The comparison of interactions among the wild-type PKCι, PKCι-F66Y and PKCι-G34W is shown in Fig. 2. The number of hydrogen bonds between PKCι and Par-6 is different for wild-type and the mutant. The comparison of the Van der Waals energy, electrostatic energy and the desolvation energy shows that the binding is altered for the mutants (Additional file 1: Table S2). These altered parameters may lead to an unstable binding between PKCι and Par-6, leading to premature separation of the two proteins. In the case of PKCι G34W, the binding between the two proteins is weaker than in the case of PKCι F66Y.

### Structural characterization of PKCι variants docked with Par-6

**Fig. 3:**
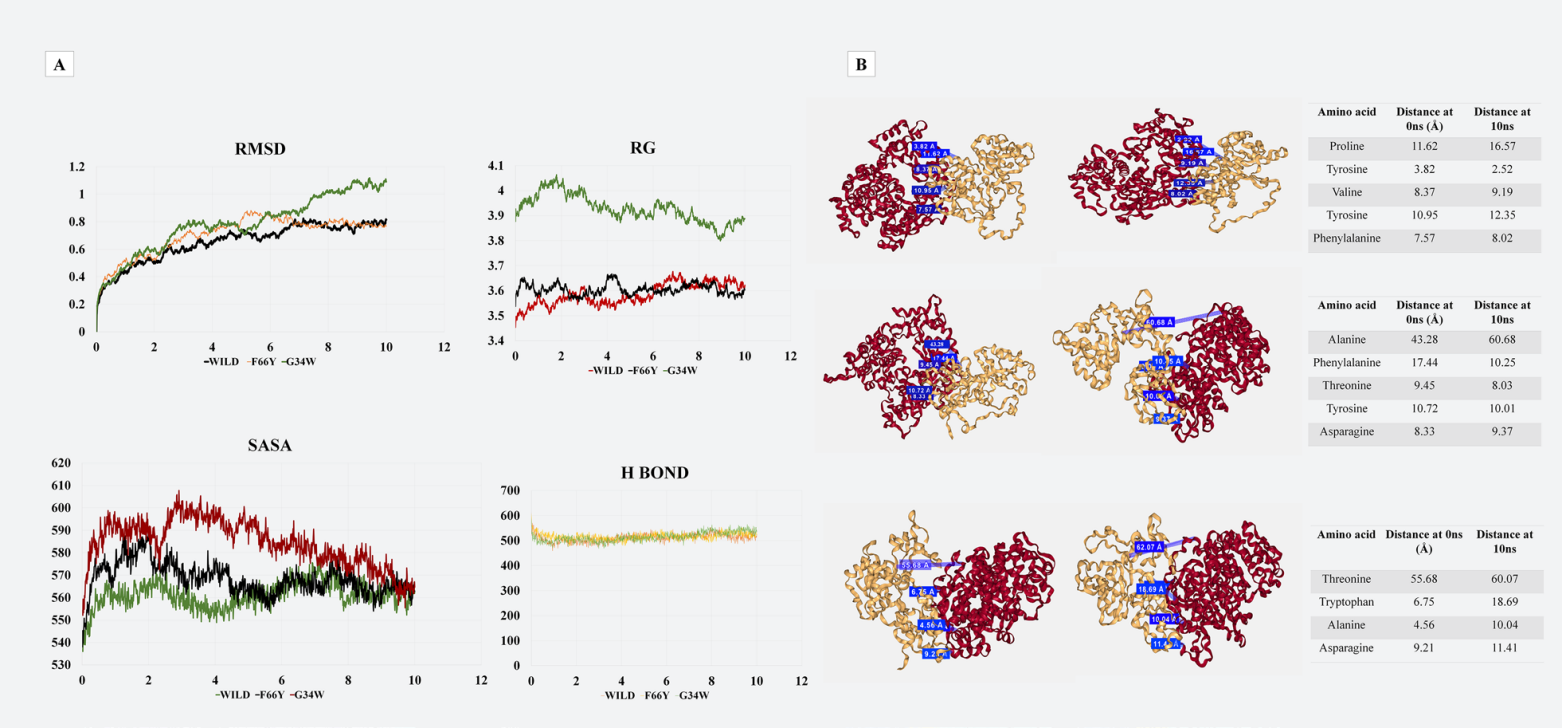
**A** Comparison of Molecular Docking simulation of PKCι wild-type and variants. Root mean square deviations (RMSD) of three docked structures. Solvent accessibility surface area (SASA) values for wild-type and mutant PKCι. Radius of Gyration comparison. Changes in the number of hydrogen bonds. **B** Comparison of the distance between chosen amino acids of Par-6 and PKCι wild-type, PKCι G34W and PKCι F66Y. The dark colored protein is the PKCΙ while the light colored is Par-6. The docked structures are visualized at 0 ns and 10 ns of the MD simulation

The degree of protein conformational changes that occur during the simulation was calculated in terms of root mean square deviation (RMSD) for wild-type and mutant docked proteins Fig. 3. The RMSD values of the PKCι wild type and F66Y are similar to each other while a clear difference in the G34W PKCι can be seen. The solvent accessible surface area (SASA) values are a measure of the accessibility of the protein to the solvent. The graph shows that the SASA values for G34W-Par-6 are higher than the wild-type, meaning that the interactions between the two proteins are weaker. The opposite is happening in the case of F66Y, indicating that surface area is lower and the protein is tightly bound. This may lend rigidity to the structure, leading to poor functional availability. The distribution of the protein atoms around its axis is the radius of gyration (Rg) and it can clearly be seen from the graph that effect of G34W on Rg is drastic. The increased value of Rg in case of G34W again indicates a weaker binding than the wild-type. Little change in terms of hydrogen bonding was observed during the simulations.

The MD simulation was visualized and pictures were captured at the beginning and end of the simulation to compare the change in the distance between some of the binding amino acids. The distances between the binding amino acids of G34W-Par-6 have the greatest difference and this coincides with results of RMSD, SASA and Rg values.

### Role of PKCι in cell polarity pathway

**Fig. 4:**
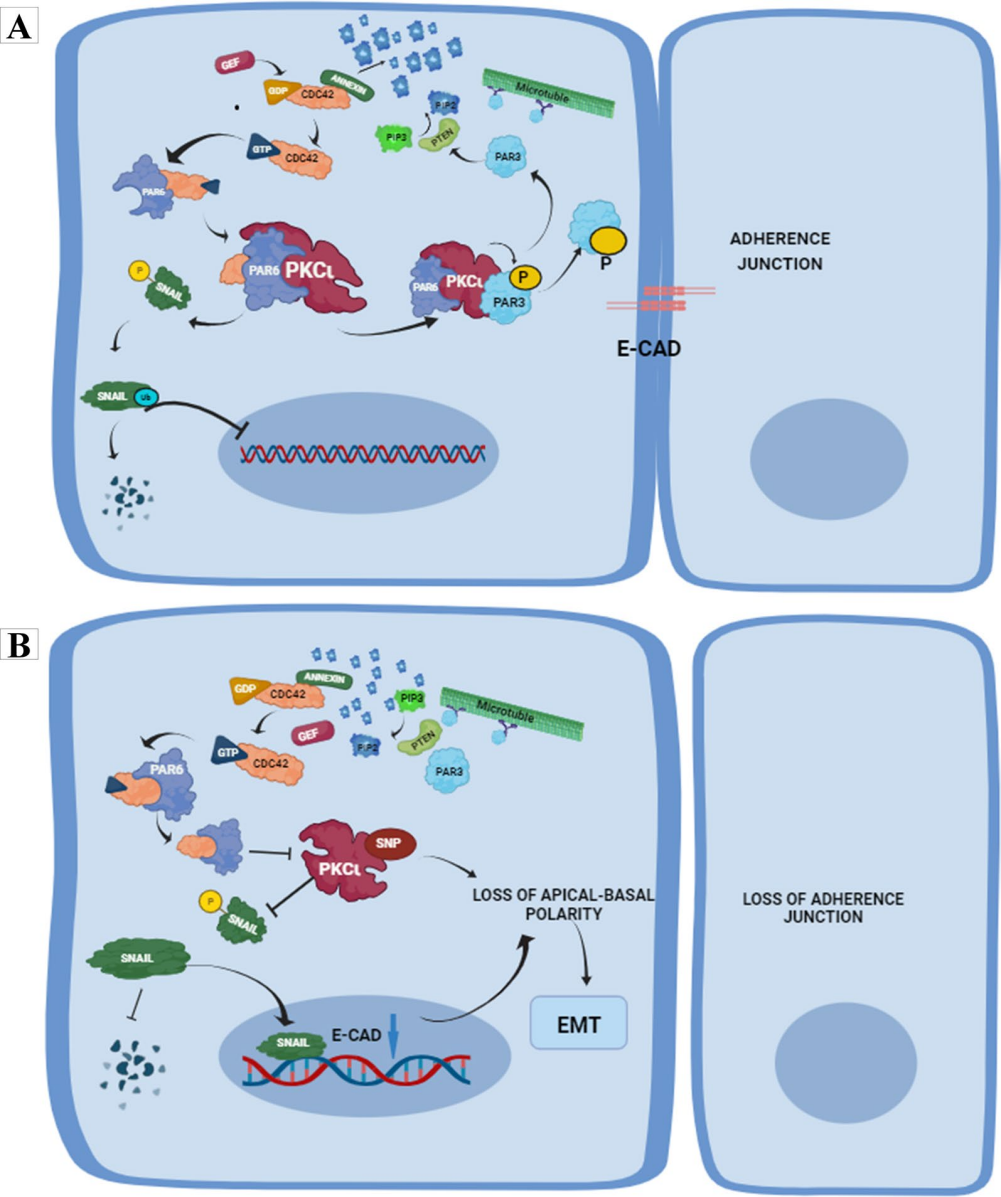
Role of PKCι in maintenance of cellular polarity. **A** PKCι wild type under normal conditions establishes a positive feedback loop through the Par complex to keep Par-3 at the adherence junctions to maintain cellular polarity. PKCι also controls the cellular concentrations of SNAI1 transcription factor by marking it for ubiquitination, in order to prevent SNAI1 from downregulating E-cadherins. **B** Structural changes in PKCι due to SNPs may lead to disturbance of the apico-basal polarity leading to EMT

The polarity pathway involving PKCι and the Par complex is shown in Fig. 4. The partitioning defective (Par) complex, consisting of Par-3, Par-6 and the atypical PKCι, plays a role in maintenance of cell polarity [23, 24]. During the establishment of epithelial cell polarity, interaction of Par-6 with PKCι localizes it to the apical region of the cell. The aberrant interaction between PKCι and Par-6 due to rs1197750201 and rs1199520604 may lead to disturbance of the feedback loop leading to loss of apico-basal polarity and epithelial to mesenchymal transition (EMT) Fig. 2. PKCι phosphorylates SNAI1 at S249 thus marking it for ubiquitination and subsequent degradation. Disturbance in the PKCι-Par-6 binding due to changes in PKCι structure as a result of polymorphism may lead to inability of PKCι to phosphorylate SNAI1, thus halting its degradation.

## Discussion

Numerous scientific investigations have revealed that several diseases, particularly malignant tumors, are associated with PRKCI as well as its various other isoforms. A recent study was conducted to investigate the effects of the PKCι missense variant in hepatocellular carcinoma. Using a variety of bioinformatics methods, the research evaluated the potentially harmful effects of the PKCι non-synonymous SNP rs1199520604 computationally. Next, using ARMS-PCR the relationship between PRKCI missense variant and HCC was evaluated. It was revealed that the heterozygous genotype of the variant seems to be protective against HCV-induced HCC, but the homozygous T genotype was found to be a risk factor. The results point to a strong disease correlation of the PKCι missense variant in the Pakistani population [25]. Another study evaluated the pathogenic potential of PKCι variants using computational analysis. This study demonstrated that the missense variants in the regulatory domain of the PKCι protein were associated with structural alterations and higher pathogenic functions [26]. A study also studies the association of PKCι missense variant with breast cancer and it was demonstrated that PKCι variant was indeed associated with breast cancer and holds the potential to serve as a possible genetic marker for breast cancer early diagnosis as well as prognosis [27]. PKCι was demonstrated to exhibit amplification in copy number in cervical cancer patients in the Chinese population. further investigation revealed that elevated expression of PKCι was associated with poor survival rates in cervical cancer patients and was related to radiation-induced proliferation alteration in cell cycle and apoptosis inhibition [28].

Therefore, there was a need to further investigate the association of PKCι variants with prostate cancer patients. The aim of this study was to determine whether PKCι missense variations G34W and F66Y have an association with PCa and whether they might serve as prognostic markers. Loss of function mutation R480C in the catalytic domain of PKCι renders the enzyme inactive, causing its inability to perform its function in polarity formation, and thus leading to malignancy [29]. Truncations and indel mutations also affect the catalytic activity of PKCι [30]. Missense mutation (R480C) in the catalytic domain along with PKCι amplification have been found in laryngeal squamous cell carcinoma (LSCC), showing a paradoxical role of PKCι, where loss of function as well as amplification are observed simultaneously [31]. PKCι over-expression has been linked with prostate cancer [32] and it was shown to promote malignancy by enhancing tumor cell motility and invasion [33]. On the basis of these data it can be hypothesized that the deleterious effects of rs1199520604 G34W can lead to loss of function for PKCι. Protein–protein interactions are essential for normal functioning of PKCι, particularly in the maintenance of apical polarity. The effect of G34W and F66Y variation on the interaction of PKCι with Par-6, which is one of the main interacting partners for cellular polarity, showed that mutant PKCι interactions may be unstable. The PKCι interacting partner Par-6 alpha has the type B PB1 domain consisting of the conserved lysine residue [7] and this interacts with the OPCA segment in the PKC PB1. Any factor that may disturb this interaction is of crucial importance. Missense mutations in the PB1 domain of PKCι can change the structure of the OPCA segment or the lysine variant which can have unpredictable effects on the interacting partners. Maintenance of cellular polarity is essential for tissue integrity, asymmetrical division [34] and apical-basal polarity of epithelial cells and defects in the complexes responsible for cell polarization can lead to pathologic epithelial-to-mesenchymal transition [35].

To observe the changes in the interaction between Par-6 and PKCι due to mutation in the PB1 domain, molecular docking with wild-type and variant PKCι exhibited a clear change in the interaction due to the missense variations. Although both variants altered the interaction of PKCι with Par-6, PKCι-G34W weakened the molecular interactions to a greater extent. The docked structure of Par-6 with PKCι wild-type, PKCιG34W variant and PKCι-F66Y variant were evaluated for their stability through molecular dynamic simulations. The reported modifications in RMSD, SASA, and Rg values provide crucial structural information about the interactions between PKCι variants (wild-type, F66Y, and G34W) and Par-6.

Establishing a clear link between these structural changes and their potential functional roles enhances the understanding of how these differences may impact protein behavior. The conformational changes in the proteins during the simulation are measured using RMSD. In comparison to the wild-type and F66Y, G34W has a larger RMSD, which indicates more structural flexibility [36, 37]. G34W's increased conformational flexibility could change the binding kinetics with Par-6 and the strength and stability of their interactions [38]. This might have an effect on the protein complex's ability to maintain cell polarity. SASA values show how much of the protein surface is exposed to the solvent. Greater exposure is indicated by higher SASA values in G34W, whilst a more compact structure is suggested by lower values in F66Y. Higher SASA suggests weaker interactions in G34W, which could lead to a reduction in binding affinity. On the other hand, F66Y's more compact shape points to a tighter binding contact, potentially influencing the functional availability of the protein complex. An increased Rg in G34W indicates a more extended structure compared to the wild-type. The drastic effect of G34W on Rg suggests a significant alteration in the overall shape and folding of the protein complex [39]. This may impact its ability to interact with other cellular components and participate in key signaling pathways related to cell polarity. While hydrogen bonding may not be the primary driver of structural changes, the persistence of certain hydrogen bonds may still contribute to the overall stability and specificity of the protein complex.

PKCι is involved in maintaining cell polarity in combination with Par-6. Any alteration in the way these proteins interact could prevent the cell polarity pathway from operating normally. Genetic variants impacting these interactions may cause a disruption in the feedback loop, which may cause apico-basal polarity to diminish, and epithelial-to-mesenchymal transition (EMT) develop. The process known as epithelial-mesenchymal transition (EMT) is linked to the growth of new tissue, the healing of wounds, and the advancement of PCa [40]. PKCι marks SNAI1 for ubiquitination and subsequent degradation by phosphorylating it at S249 [41]. Genetic variants in PKCι may cause SNAI1 to accumulate due to alterations in SNAI1 and PKCι interaction and potentially impair cellular functions regulated by SNAI1, such as cell invasion and migration [42], if they impact this phosphorylation process through changed interactions among PKCι-Par-6.

The changes in the mRNA secondary structure upon variations are in contrast with above mentioned results, showing rs1199520604 (G34W) as stable while rs1197750201 (F66Y) secondary structure was unstable as compared to wild-type due to higher MFE. The secondary structure of mRNA can have a noticeable effect on protein structure as well as on its interactions with other binding partners. Studies have shown that the stable mRNA secondary structure tends to impact the translation of proteins. Highly stable mRNA hamper the accessibility of ribosomes at the translation start site resulting in reduced translation rates. This slower process of translation can impact the co-translational folding of the newly synthesized polypeptide chain, ultimately affecting its tendency to interact and bind with its other binding proteins [43]. The stability and appropriate folding of the translated protein can also be impacted by the stability of the mRNA structure. Maintenance of protein-interaction domains and fostering of molecular interactions with different interacting proteins depend on proper protein folding. The structure and interaction potential of the protein can be impacted by translation-induced instability due to the highly stable secondary structure of mRNA [44, 45].

In terms of genotypic analysis, PKCι variant rs1199520604 (G34W) genotype TT had the most significant association with increased risk of prostate cancer. These results are consistent with other results of G34W variant, as it was shown to be responsible for drastic changes in PKCι interactions with Par-6. PKCι F66Y on the other hand did not show potential as a prognostic marker for PCa, as the wild-type genotype had the strongest association with elevated risk of PCa.

The identification of rs1199520604 (G34W) as a putative prognostic marker raises the possibility that individuals who carry this genetic variant will experience their illness differently from those who do not. It could be a sign of altered risk of disease events, such the course of the disease, its recurrence, or how well a treatment works. The development of genotypic analytic methods to detect the existence of rs1199520604 and other pertinent genetic variations in patients with prostate cancer is one of the potential implications. More focused and efficient interventions could be made possible by using this genotypic data to particularly design treatment plans for people who carry this genetic variation. With this data, techniques for personalized medicine can be put into practice, such as tailored diagnostic methods, prognosis evaluations, and treatment regimens based on the unique genetic profile of each patient [46]. Potential therapeutic targets can be identified by comprehending the molecular interactions and pathways impacted by genetic differences. Subsequent investigations may delve into developing focused treatments intended to reinstate or adjust the disturbed associations between PKCι and Par-6, which could potentially impact cellular polarity, the EMT, and the advancement of cancer [41, 47]. The study highlights PKCι's possible role in EMT, a phenomenon linked to the advancement of cancer. Further studies may explore the precise pathways by which PKCι impacts epithelial-mesenchymal transition, offering perspectives into the more extensive cellular and molecular mechanisms implicated in PCa metastasis.

In a wider perspective, the results of this study open up the possibilities for personalized medicine strategies for the treatment of prostate cancer, wherein prognostic and diagnostic tools could also incorporate genotypic study of PKCι genetic variations. The discovery of particular genetic markers linked to prostate cancer (PCa) creates opportunities for the development of precision medicine interventions and targeted therapeutics. Furthermore, comprehending the molecular dynamics of PKCι interactions may aid in the development of innovative therapeutic drugs targeted at modifying epithelial cell polarity pathways and, as a result, preventing the advancement of cancer. Therefore, these genetic variants hold a significant potential for further analysis through in vivo as well as in vitro investigations. Finally, to gain a deeper understanding of these genetic variations' molecular functions, it is essential to carry out extensive functional studies on a sizable cohort. These studies seek to clarify the complex processes that underlie these variations, opening the door to their possible application as cutting-edge targets for therapy for the treatment of PCa. Furthermore, the identified molecular markers could establish these variations as novel biomarkers, hence supporting improved prognosis and early detection approaches for PCa.

## Conclusion

In conclusion the study highlights that SNPs rs1199520604 (G34W) and rs1197750201 (F66Y) might serve as a prognostic marker for PCa because of significant association with PCa. In silico evaluation of changes in PKCι molecular interactions due to rs1199520604 (G34W) also indicated impaired interactions with Par-6 also strengthens its role as a prognostic marker.

## Limitations

In vitro and in vivo studies involving larger data sets are required to further validate these results. The effect of chosen polymorphisms on protein structure also need to be validated through wet lab analysis. The effect of missense variants on PKCι downstream function and interactions also need further exploration.

### Supplementary Information

**Additional file 1.** Supplementary Figures and Tables.

## Data Availability

All data generated or analyzed during this study are included in this article.
