# Supplementary material for: Possible prognostic impact of PKCι genetic variants in prostate cancer
Source: Cancer Cell Int. 2024 Jan 10;24:24. doi: 10.1186/s12935-023-03182-4 (PMC10782671; doi:10.1186/s12935-023-03182-4)
Supplement: Supplementary file 1 — Additional file 1. Supplementary Figures and Tables. [file 12935_2023_3182_MOESM1_ESM.pdf]

Supplementary File1

Possible Prognostic Impact of PKCι Genetic Variants in Prostate Cancer.

Amna Hafeez[1], Maria Shabbir[1,] Khushbukhat Khan[1], Janeen H. Trembley[2,3,4], Yasmin Badshah[1], Sameen Zafar[1], Kanza Shahid[1], Hania Shah[1], Naeem Mahmood Ashraf[5], Arslan Hamid[6], Tayyaba Afsar[7], Ali Almajwal[7,] , Suhail Razak[7]

Table 1: PRKCI primer sequences for Tetra-ARMS PCR

**PRKCI primers for Tetra-ARM's PCR**		
**G34W (rs1199520604)**	Forward inner	5'-GTGAAAGCCTACTACCACG-3'
	Reverse inner	5'-TCCCAGGACACTCATCA-3'
	Outer forward	5'-AGGTGGGCAGGTAGGT-3'
	Outer reverse	5'-CACCCCTATCACTTCGTC-3'.
**F66Y (rs1197750201)**	Forward inner	5' -GTGTTCTTTTGACAACGAACAGCTGT-3'
	Reverse inner	5'-CTTCCTCATCTATCCATTTCATGGAGA-3'
	Forward outer	5'- AAGTTCATCAAATTGTCAAGCATTCAGT-3'
	Reverse outer	5'-AAGAAAAAACAAGAATGGCATCAAATTT-3'

Table 2: HADDOCK results for the docking interactions

**Interaction**	**Wild-Type PKCI-Par-6**	**PKCI-F66Y-Par-6**	**PKCI-G34W-Par-6**
HADDOCK score	56.7 +/- 13.0	90.5 +/- 16.3	15.6 +/- 26.0
RMSD from the overall lowest-energy structure	21.7 +/- 0.7	22.0 +/- 0.3	0.8 +/- 0.5
Van der Waals energy	-81.7 +/- 7.4	-96.3 +/- 7.0	-79.6 +/- 8.3
Electrostatic energy	-167.6 +/- 46.1	-184.8 +/- 7.3	-182.0 +/- 34.6
Desolvation energy	-14.5 +/- 6.3	-30.0 +/- 2.8	-36.4 +/- 3.5
Buried Surface Area	2803.2 +/- 269.5	3635.1 +/- 100.9	2970.3 +/- 38.1
Z-Score	-2.0	-1.7	-1.8
